# Vanishing sea ice threatens Southern Ocean isopod species diversity

**DOI:** 10.1098/rsbl.2026.0309

**Published:** 2026-11-04

**Authors:** Davide Di Franco, Katrin Linse, Huw Griffiths, Angelika Brandt

**Affiliations:** ^1^ Senckenberg Research Institute and Natural History Museum, Frankfurt am Main, Germany; ^2^ Institute for Ecology, Diversity and Evolution, Goethe University Frankfurt, Frankfurt, Germany; ^3^ Paleontology and Collections, GeoSphere Austria, Vienna, Austria; ^4^ British Antarctic Survey, Cambridge, UK

**Keywords:** Isopoda, climate change, biodiversity loss, Weddell Sea, sea-ice loss impact, Southern Ocean benthos, environmental variables, physical drivers

## Abstract

Sea-ice retreat is rapidly transforming the Southern Ocean (SO), yet consequences for benthic biodiversity remain poorly resolved. Here, we show that declining sea ice is directly linked to reduced species diversity in benthic isopods, a dominant SO fauna (Pearson's *r* = 0.56). We analysed 214 isopod species from 78 genera in the Atlantic sector using a 14-year dataset integrating satellite and *in situ* observations across 60–77° S and 403–4976 m depths. Of these 214 species, 32% (68) occur exclusively at high sea-ice stations, and among them 53% are new to science and 75% are rare. Redundancy analysis (variance explained 15.3% and 6.5% for axes 1 and 2; *p* ≤ 0.002) and generalized linear models (*p* = 0.0025) identify sea-ice cover as a primary driver of species richness (Pearson's *r* = 0.56) and community composition. Declining sea ice is associated with lower diversity and shifts in assemblage structure, indicating strong coupling between cryosphere change and deep-sea biodiversity. Given that the SO is a global biodiversity hotspot, these findings reveal unquantified vulnerability of seafloor communities. With continued sea-ice decline projected, irreversible biodiversity losses are likely, underscoring an urgent need for climate mitigation as impacts reach the farthest marine frontiers.

## 1. Introduction

Despite the low temperatures and polar environment, the Southern Ocean (SO) hosts a unique fauna with high levels of diversity and endemism (more than 8000 valid marine species discovered, 50–97% of which are endemic) [1,2]. These characteristics are the result of an evolution in isolation for more than 20 Myr, and strong influence by geologic and climatic events over the last 40 Myr [3]. Extreme temperatures and oceanographic features such as the Antarctic Circumpolar Current (ACC) have contributed to this isolation by acting as barriers to most marine organisms [4]. Owing to this evolutionary history, several SO benthic taxa have developed peculiar and habitat-specific adaptations such as cold tolerance, stenothermy, slow metabolic rates, long generation times and brooding as reproductive strategy [5–7]. Precisely because of these narrow physiological tolerances and specialized adaptations, these cool-adapted taxa are considered to be particularly vulnerable to rapid changes in their physical environment [7,8].

Among SO environmental variables, sea ice, is one of the main physical drivers affecting life on the seafloor [9], which is home to about 90% of the marine species known from the region [2]. The variability in sea-ice duration and the seasonal inputs of high quantities of food particles in the SO, led to a niche diversification where benthic species adopted different feeding habits in relation to sea-ice extension and persistence. For instance, up to 95% of Antarctic species that directly consume phytoplankton or benthic algae cease to feed in winter owing to the reduced availability of suitable food sources [6]. Many suspension-feeders that feed throughout the year shift their food target from large particles to smaller ones owing to seasonal changes in food availability [6]. The warming-induced reduction of sea-ice coverage and alteration of its seasonality owing to climate change will make vertical food transfer available for longer periods and alter its composition [10]. In addition, a decline in sea-ice extent will lead to a decrease of the overall sea-ice algae abundance and reduce the frequency of ice-related phytoplankton blooms, shifting species composition towards smaller diatoms and flagellates [10]. This will force benthic taxa to change their feeding habits and/or find alternative food supplies, leading to taxonomic shifts in their communities, migration and even extinction events for those species that are unable to adapt [11–13]. Even in the deep sea, major changes (with potential ocean temperature increase by 1°C over the next 84 years and a decline in O_2_ concentration in the SO of up to 3.7% or more) are expected to affect deep benthic species and ecosystems, however, these ecosystems are very poorly understood [14]. Sediment inputs through glacier melting affect coastal ecosystems on the Antarctic Peninsula as well as carbon flows in Antarctic benthic food webs [15,16]. However, effects on deep-sea faunal elements have not yet been studied. Therefore, our study aims to expand knowledge on the diversity of deep-sea benthic isopods from SO areas characterized by different environmental conditions, including the southernmost Filchner Trough, by investigating the influence of environmental variables on species richness and assemblage composition.

## 2. Material and methods

The study is based on isopod samples (figure 1A) collected using an epibenthic sledge (EBS) [17] during four different expeditions conducted between 2012 and 2019 in the Atlantic sector of the SO: JR275, JR15005 (figure 2A–F), JR17003a on board the RRS *James Clark Ross* and PS118 expedition on board of RV *Polarstern* [18]. Samples were collected in the Atlantic sector of the SO between 60° S and 77° S and at depth ranges between 403 and 4976 m. For comparability, abundance data were standardized to 1000 m haul distances. A total of 16 157 isopod specimens were preserved on board in 96% ethanol and subsequently identified and counted in the laboratory. To evaluate the robustness of our findings for biodiversity patterns in the SO deep sea, we included a further 22 epibenthic sledge samples collected during ANDEEP (ANtarctic benthic DEEP-sea biodiversity, colonization history and recent community patterns) expeditions from bathyal and abyssal depths of the study region (figure 1B). Environmental data were downloaded from the ‘global environmental datasets for marine species distribution modelling’ Bio-ORACLE (http://www.bio-oracle.org/) [19] with a resolution of 5 arcminutes. Bio-ORACLE data are based on a combination of satellite and *in situ* observations, for a 14-year period (2000–2014). Layers downloaded included long-term annual mean values (averaged across all months over the entire 14-year period) at the maximum depth recorded of salinity, temperature (°C), chlorophyll-a (mg m^−3^), silicate (mol m^−3^), current velocity (ms^−1^), iron (µmol m^−3^), oxygen concentration (mol m^−3^), nitrate (mol m^−3^), primary production (g m^−3^ d^−1^), phosphate (mol m^−3^), phytoplankton biomass (µmol m^−3^), sea-ice concentration (fractions from 0.1 to 1, where 0.1 indicates very low concentration/absence of sea ice and 1 indicates a completely ice-covered area). Environmental variables were checked for collinearity using a Draftsman plot analysis based on Pearson correlation coefficient through the software PRIMER 6 [20]. According to the Pearson correlation coefficient, the following variables were removed: silicate, nitrate, phosphate, phytoplankton biomass. The correlation between isopod species and environmental variables was investigated by means of Pearson correlation analyses using the statistical software RStudio and the package ‘ggpubr’ [21]. A redundancy analysis (RDA) and a generalized linear model analysis (GLM) were performed on square root transformed species abundance data to assess the physical driving variable shaping isopod species community composition and species richness, respectively. Before analyses, environmental variables were normalized for comparability. A detrended correspondence analysis (DCA) was performed to assess whether taxa responses were best explained by unimodal or linear models, thus to evaluate whether a Hellinger transformation was needed [22,23]. DCA analysis showed that the gradient length of the first axis was greater than 2 (5.25 SD), therefore Hellinger transformation was performed on the dataset to perform the RDA. In addition, a Monte Carlo permutation test was performed to test the significance of the variation in species composition explained by environmental variables and identify the most significant axis/axes (999 permutations). GLMs analysis was initially fitted with a Poisson distribution, in case of over-dispersion the model was subsequently fitted with a quasi-Poissonregression. To check for over-dispersion, the library ‘qcc’ for RStudio was used [24]. While the RDA analysis was done using the library ‘vegan’ on RStudio [25], the GLMs analysis was done using the function ‘glm ()’ on the same software. Species records and environmental data are available from the Polar Data Centre under Di Franco *et al.* [26].

**Figure 1.**
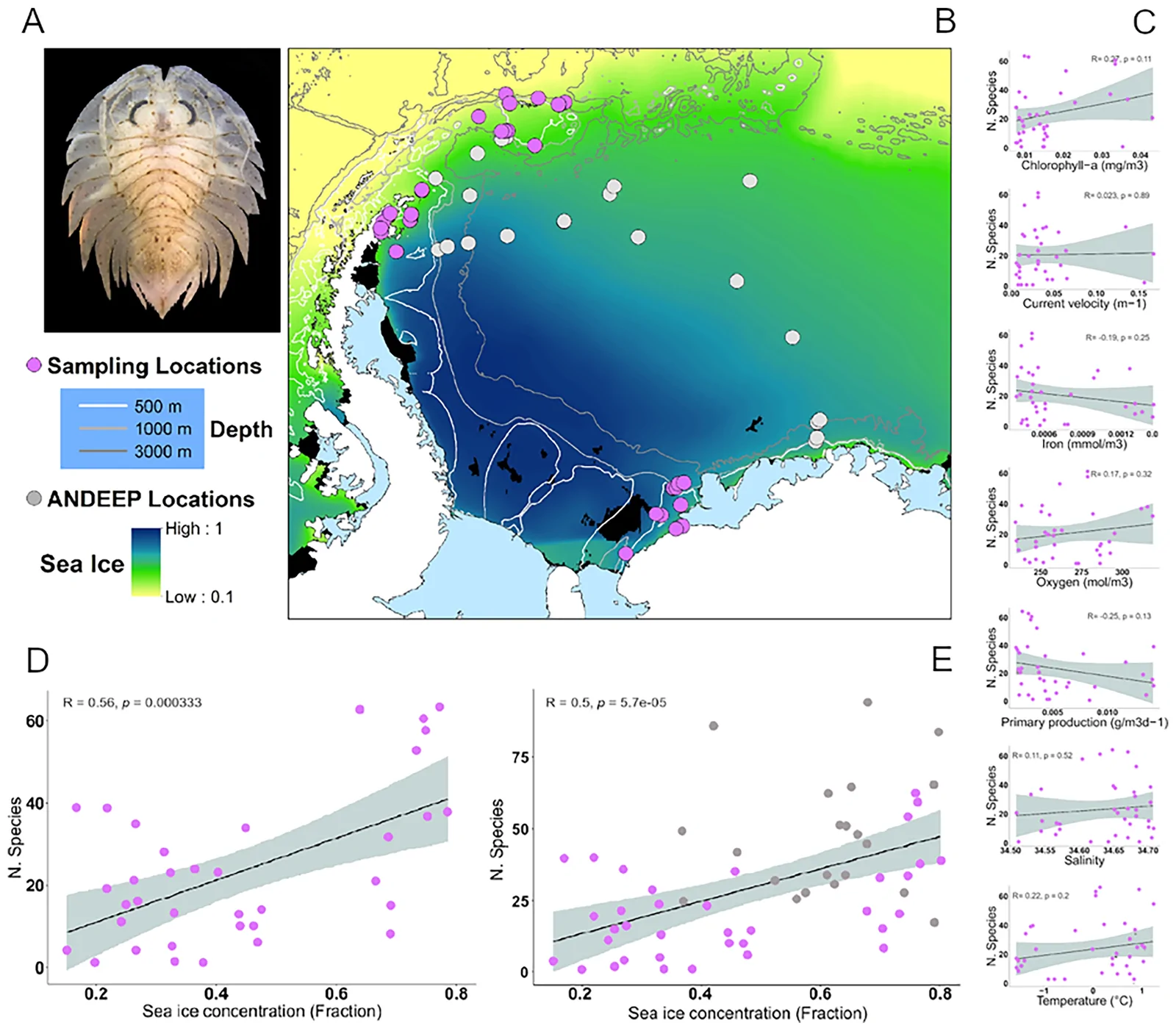
Correlation between sea-ice concentration and benthic isopod in the Southern Ocean (SO). (A) The exemplary benthic isopod species *Ceratoserolis trilobitoides* (Eights, 1833) from the Weddell Sea (SO). (B) Map of the study area, pink circles show stations from the shelf and upper slope (expeditions JR275, JR15005, JR17003a and PS118); grey circles are stations from the deep-sea ANDEEP II-III expeditions; ice concentration is given using the unit interval (fractions) from 0.1 to 1, where 0.1 indicates very low concentration/absence of ice and 1 indicates a completely ice-covered area. (C) Pearson correlation analysis of environmental variables and isopod species number from the shelf and upper slope. (D) Pearson correlation analysis of sea-ice concentration and isopod species number from the shelf and upper slope. (E) Pearson correlation analysis of sea-ice concentration and isopod species number from the shelf, upper slope (pink circles) and deep-sea ANDEEP II-III expeditions (grey circles).

**Figure 2.**
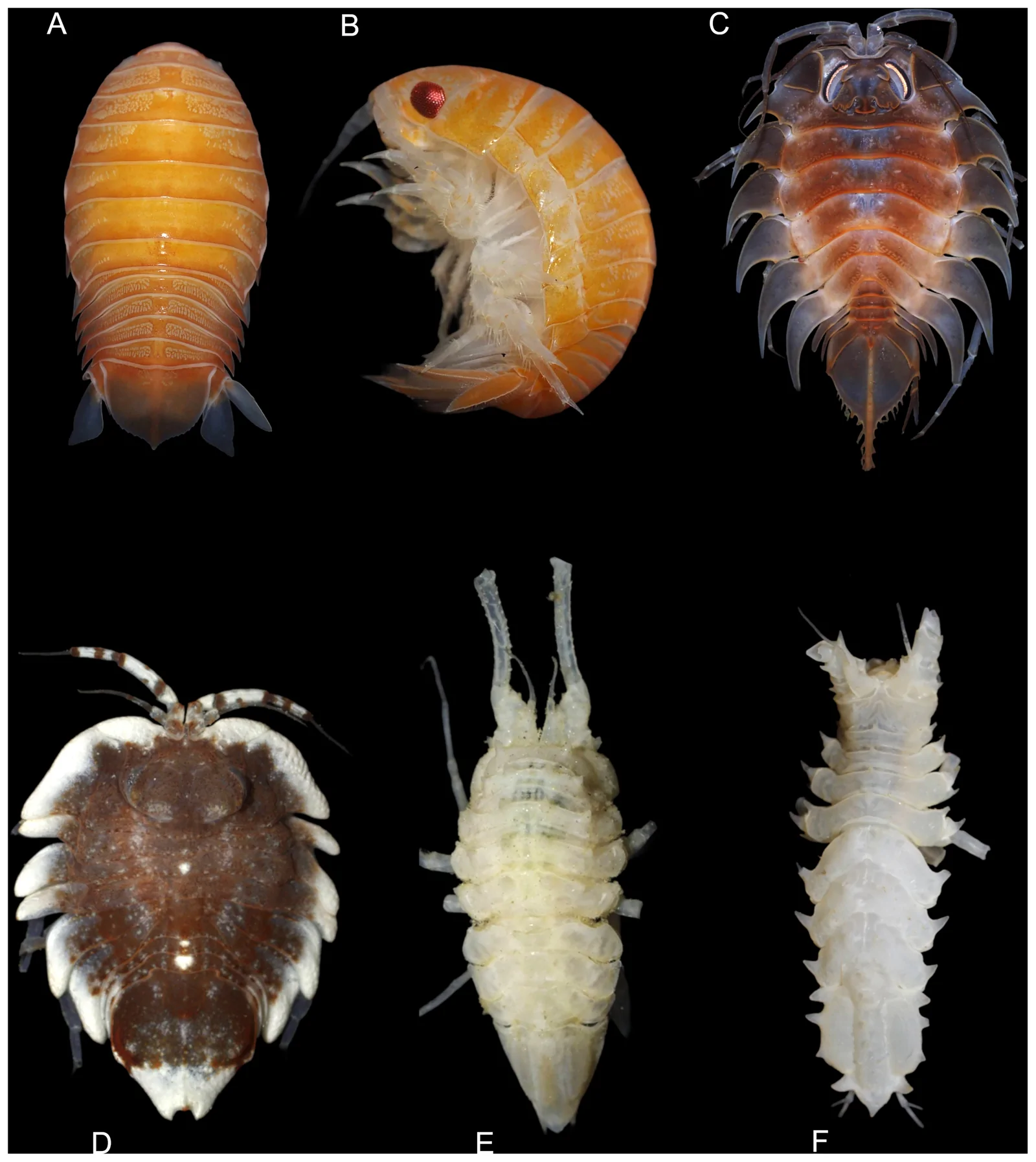
Representative Southern Ocean isopods collected during expedition JR15005. (A) *Natatolana* sp., (B) *Natatolana cf. oculata* (Vanhöffen, 1914), (C) *Ceratoserolis meridionalis* (Vanhöffen, 1914), (D) *Serolella bouvieri* (Richardson, 1906), (E) *Notopais* sp., (F) *Vanhoeffenura* sp. Photo credit: JR15005 expedition participants.

## 3. Results and discussion

The material analysed for our study comprised 37 epibenthic sledge samples collected on the shelf and upper slope of the South Atlantic sector of the SO (including the Weddell Sea) (figure 1B), comprising a total number of 214 species.

Among the 214 species recorded, 32% (68 species) were exclusively found in sampling stations characterized by higher sea-ice concentrations (more than 0.5 fraction); within this exclusive group of 68 species, 36 species (53%) are species new to science and 51 species (75%) are rare species characterized by very limited geographic distributions, including endemics. Notably, there is some overlap between these two latter categories, as most of the newly discovered taxa present a limited geographic distribution. While the rarity of some taxa might be partially influenced by sampling artefacts typical of deep-sea environments (e.g. sampling bias), the pronounced endemism and specialization of the Southern Ocean benthos suggest a substantial vulnerability [1,7,11]. Stations with higher sea-ice concentration (more than 0.5 fraction) presented a higher number of specialist species, e.g. four different species of the selective phytodetritivore family Macrostylidae Hansen, 1916 and seven selective deposit-feeding species belonging to the family Ischnomesidae Hansen, 1916, while only one species of Ischnomesidae and no Macrostylids were found at stations of lower sea-ice concentrations. Locations with higher sea-ice concentrations showed a higher abundance of specimens of the specialist, sponge-commensal family Dendrotionidae Vanhöffen, 1914 compared to stations with low sea-ice concentration (137 and 7 specimens, respectively). This might be explained by a possible correlation with higher abundances of sponges in areas with higher sea-ice concentrations. A longer sea-ice duration was found to be positively correlated with the percent cover of sponges in seasonally ice-covered areas [27]. In our study, areas with higher concentrations of sea ice and especially those in the southeast Weddell Sea showed the highest number of rare species and species previously known only from their type locality.

### (a) Sea-ice influence on isopod species richness and composition

While salinity, temperature, chlorophyll-a, current velocity, iron, oxygen and primary production did not show any correlation with isopod diversity (figure 1C), the number of isopod species increased with increasing sea-ice coverage (figure 1D).

The RDA analysis identified current velocity and sea ice as the environmental variables that best explained the variation in isopod species composition (table 1). Canonical axes explained 41.73% of the variance and the permutation test showed that the first and second axes were the most significant (*p* = 0.001 and *p* = 0.002, respectively). These latter explained 15.3% and 6.5% of the variance, respectively (table 1).

**Table 1.** RDA analysis on benthic isopod species from the continental shelf and slope of the Weddell Sea.

partitioning of variance						
	inertia proportion					
total	0.8085	1				
constrained	0.3374	**0.4173**				
unconstrained	0.4711	0.5827				
eigenvalues and their contribution to the variance						
importance of components						
	RDA1	RDA2	RDA3	RDA4	RDA5	RDA6
eigenvalue	**0.1234**	**0.05261**	0.04007	0.02633	0.02267	0.0224
proportion	explained	0.1527	0.06508	0.04956	0.03256	0.02804
cumulative	proportion	0.1527	0.21777	0.26733	0.29989	0.32793
biplot scores for constraining variables						
	RDA1	RDA2	RDA3	RDA4	RDA5	RDA6
depth	−0.29229	−0.12082	0.22178	0.4524	−0.28958	−0.55053
Chl-*a*	−0.32279	0.56316	−0.18915	0.28004	0.17979	0.50116
cv	**−0.50894**	−0.279	0.41653	−0.41117	0.03733	−0.42775
O_2_	−0.08966	0.58446	−0.60217	−0.04533	0.27598	0.16345
Fe	0.22983	0.14628	−0.54425	−0.11074	0.31719	0.34537
pp	0.35732	0.12019	0.07915	0.13504	0.24813	0.30047
sal	−0.0979	−0.05678	−0.26161	0.01151	0.23913	0.31883
temp	−0.33216	−0.37322	0.54346	0.01639	−0.36351	−0.01977
ice	−0.21434	**0.65379**	−0.39022	0.16664	−0.43679	0.2141

Abbreviations: Chl-*a* = chlorophyll-a, cv = current velocity, O_2_ = oxygen, Fe = iron, pp = primary productivity, sal = salinity, temp = temperature, ice = sea-ice concentration. Bold values indicate the highest biplot scores for the first and second RDA axes.

The GLMs model showed sea ice (*p* = 0.0025, 23.2% of deviance explained) and chlorophyll-a (*p* = 0.011, 15.0% of deviance explained) as the main variables driving species richness variations among sampling sites. The model was first fitted with Poisson distribution, however the latter indicated over-dispersion of the data (over-dispersion test *p* < 0.05), therefore the model was subsequently fitted with a quasi-Poisson regression.

The influence of sea-ice concentration on isopod species richness and composition shown in these results is explained by sea-ice dynamics and its seasonal break-up, an important result, as these dynamics on ecosystems are not very well understood [14]. When sea ice melts, large quantities of sympagic algae and animals are released, triggering phytoplankton blooms and thus enhancing the productivity in the water column and the amount of organic matter available for the benthos [28–30]. In addition, sea-ice melting processes release freshwater, increasing the stratification of the water column that in turn enhances the amount of light availability and thus primary productivity [31,32].

When analysing the expanded dataset including the ANDEEP samples (totalling 763 nominal species), the positive correlation between species richness and sea-ice concentration was confirmed (figure 1E). Especially the southeast Weddell Sea, a region characterized by high sea-ice concentration, had a much higher diversity of isopods than the northern region around the Antarctic Peninsula. GLM analysis indicated sea-ice concentration (*p* < 0.001, 25.1% of deviance explained), iron (*p* = 0.002, 17.7% of deviance explained), primary productivity (*p* = 0.004, 14.8% of deviance explained) and depth (*p* = 0.003, 14.5% of deviance explained) as the main environmental variables driving species richness between sampling stations. The influence of iron on species richness is related to the aforementioned sea-ice dynamics. Large quantities of iron accumulate in sea ice and are released when this latter breaks up and melts; sea ice is considered as the dominant source of iron in Polar waters during seasonal melting [33]. In the Southern Ocean, the growth of phytoplankton is strongly limited by the availability of iron [33,34], thus large pulses of seasonal iron inputs through sea-ice melting highly increase the productivity in the water column [35]. Despite the low sedimentation rate that characterizes the deep sea [36–38] and the reduced quantity of organic matter reaching the seafloor [39,40], it has been shown that deep-sea benthic communities from abyssal depths can quickly react to phytodetritus pulses [41–43]; although other studies did not show a clear response [36,44] or showed that benthic functionality changes [45]. It has been assumed that deep-sea isopod species probably present an opportunistic feeding strategy and mainly rely on seasonal pulses of fresh phytodetritus on the seafloor [46]. Moreover, in the Southern Ocean the number of isopod species increases with depth, reaching the highest value of diversity at about 2000–4000 m [47].

A recent study identified sea-ice concentration as the main driver shaping the composition and abundance of SO peracarid orders [48]. Our new findings expanded these analyses and identified for the first time the link between species richness and sea ice in the SO, highlighting the important role that sea ice plays as driver of isopod diversity. Higher diversity and number of rare/specialist isopod species found in areas with higher sea-ice concentrations provide valuable insight to predict the consequences that a reduction in sea-ice extent would have on SO benthic communities. In addition to its role in shaping primary production and food availability, sea ice can influence benthic ecosystems through physical disturbance processes such as ice scouring, which alters seabed characteristics and habitat structure [45]. At the same time, other environmental factors such as depth, substrate type and habitat heterogeneity may also play an important role in structuring benthic communities at regional scales and in shallower waters [45].

Changes in sea-ice extent strongly affect the benthic fauna living underneath, owing to changes in food availability and food-web structure [9,27,49]; such changes may disadvantage highly specialized taxa while favouring more generalist or opportunistic species, as sea-ice loss modifies resource availability, increases environmental variability and alters trophic pathways [50]. Increased abundances and biomass associated with reduced sea-ice cover may also influence species richness, as predicted by ecological theory linking productivity and diversity [51,52]. In this context, higher productivity may alter the composition and balance of the benthic community, excluding species better adapted to the sea-ice zone [51,52].

The high isopod richness and abundance observed in the investigated regions characterized by high sea-ice concentrations (e.g. the Filchner Trough area), are probably sustained not only by local sympagic primary production but also by ocean currents [53]. The transport of organic matter and nutrients from highly productive polynyas and marginal ice zones via water masses such as warm deep water (WDW) and modified warm deep water (MWDW), plays a key role in feeding deep-sea communities [53]. Future modifications induced by climate change in sea-ice cover (including alteration or reduction of polynyas and marginal ice zones) and deep-ocean circulation threaten to alter these essential trophic links. Reduced sea ice may initially increase light availability, however shifts in sea-ice seasonality are likely to alter the quality and timing of organic matter inputs [9,27]. Deep-sea isopod species are very limited in their mobility, owing to their brooding reproductive strategy (lack of pelagic larvae), low metabolic rates and stenothermy [7,54]. As a consequence, their capacity to migrate toward more favourable regions in case of alterations of the food input is extremely limited, putting vulnerable deep-sea benthic hotspots at high risk of biodiversity loss.

The prominent influence of sea ice on benthic communities was recently reported by Pineda-Metz *et al.* [5]: the negative correlation between sea ice and primary productivity directly influences the abundance and biomass of the benthos, at decreasing levels of sea-ice coverage and increasing primary productivity abundances and biomass increase with a total gain of blue carbon. In 2021, Lin *et al.* showed the decline of plankton diversity with reduced sea-ice extent in a study that investigated at genus level a 5-year dataset of seven different genera of phytoplanktonic diatoms from the Western Antarctic Peninsula [55]. However, our findings expand these analyses to the benthic ecosystem and to species level, investigating with the Atlantic sector of the Southern Ocean a much larger area. In contrast to the pelagic ecosystem where short-time environmental changes cause shifts in community composition [56], the benthic species are less affected by short-term changes in the overlying water column. The signal of isopod diversity declines with reducing ice concentrations in benthos is very compelling and concerning. Our results suggest that a reduction in sea-ice concentration would highly threaten the striking diversity of SO benthic isopod communities.

Future projections of climate warming suggest that this strong decline in sea ice will eventually affect the entire SO, threatening both regional and global biodiversity, owing to the high levels of endemism in the SO [57]. In a warming climate scenario (RCP 8.5), the SO could lose half of its sea-ice cover by 2100 [58] and the Eastern Weddell Sea could record the greatest sea-surface temperature anomaly with respect to preindustrial values (up to 3°C by 2100) [59]. More recently, despite the gradual increase of sea ice in the Weddell Sea since the early 1980s, long-term trends showed large multi-year variability, with a recent strong decline beginning in 2016 [31,60]. Our findings suggest that this warming and loss of sea ice clearly endangers the biodiversity of the fauna living on the Weddell Sea continental shelf and in the deep sea as also documented for the West Antarctic Peninsula [45].

Almost half of the sampling locations in the extended dataset including ANDEEP samples were from bathyal and abyssal depths (between approx. 1500 and 4900 m), suggesting that the retreat of sea ice can impact even the deepest parts of the SO. Changes are currently affecting the deep sea, the mean warming rate of the deep Weddell Sea below 2000 m is five times that of the global ocean [61]. A possible reason to this trend is the variation in sea-ice coverage which can modify the heat loss from ocean to atmosphere and alter radiative fluxes. Recent studies showed the link between sea ice and deep-ocean circulation [62] and identified water masses and currents as important physical drivers affecting SO benthos [53,63–65]. In the SO, the ANDEEP expeditions showed high level of isopod species diversity down to 5000 m and levels of diversity between 500 and 2000 m similar to those present in many temperate and tropical slopes in the Southern Hemisphere [54]. The SO is a hotspot of biodiversity, it hosts 5% of the global marine biodiversity, despite representing only the 8% of global ocean surfaces [66]. Some of the SO marine invertebrates highly contribute to the world´s species richness, for example SO holothurians represent 10% of the world´s holothuroid species diversity, SO pycnogonids are probably the most species-rich on the Planet, with 20% of their total known species found exclusively in Antarctic and/or Sub-Antarctic waters [67,68] and climate change might disrupt the core habitats of species living there [69]. SO benthic fauna shows remarkable level of endemism, for instance 70% of gastropods and pycnogonids and 88% of peracarid amphipods are endemic of the SO [66,70]. In this latter, the main area investigated in this study (the Weddell Sea) is the region with the highest level of endemism [66]; owing to climate change and its consequent warming, 79% of SO endemic species face a reduction in suitable temperature habitat [11]. Ultimately, it is worth mentioning that the SO diversity is probably still underestimated, for example more than 85% of the isopod species sampled during the ANDEEP expeditions in the SO deep-sea were new to science [54]. In light of this and of our findings, it is possible to conclude that the remarkable species richness of the continental shelf and deep sea at the highest latitudes in the SO is highly threatened by climate change and the consequent loss of sea ice.

## Data Availability

The metadata for the expeditions JR275, JR15005 and JR17003a, the environmental data and the biodiversity data for the analyses of this study are deposited at the UK Polar Data Centre [26]. These metadata include geographic coordinates, depth and sampling gear details (epibenthic sledge). Furthermore, it contains the biodiversity abundance data (species counts per station) for the 214 benthic isopod species analysed, alongside the corresponding Bio-ORACLE environmental variables extracted for each sampling location.

## References

[1] Chown SL , Clarke A, Fraser CI, Cary SC, Moon KL, McGeoch MA. 2015 The changing form of Antarctic biodiversity. Nature 522, 431–438. (doi:10.1038/nature14505) 26108852

[2] De Broyer C , Danis B. 2011 How many species in the Southern Ocean? Towards a dynamic inventory of the Antarctic marine species. Deep Sea Res. Part II 58, 5–17. (doi:10.1016/j.dsr2.2010.10.007)

[3] Lawver LA , Gahagan LM, Dalziel I. 2011A different look at gateways: Drake Passage and Australia/Antarctica. In Tectonic, climatic, and cryospheric evolution of the Antarctic Peninsula (eds Anderson JB, Wellner JS), pp. 5–33. Washington, DC: American Geophysical Union. (doi:10.1029/2010SP001017)

[4] Thatje S , Hillenbrand C-D, Larter R. 2005 On the origin of Antarctic marine benthic community structure. Trends Ecol. Evol. 20, 534–540. (doi:10.1016/j.tree.2005.07.010) 16701431

[5] Pineda-Metz SEA , Gerdes D, Richter C. 2020 Benthic fauna declined on a whitening Antarctic continental shelf. Nat. Commun. 11, 2226. (doi:10.1038/s41467-020-16093-z) PMC7203119 32376915

[6] Peck LS . 2018Antarctic marine biodiversity: adaptations, environments and responses to change. In Oceanography and marine biology (eds SJ Hawkins, AJ Evans, AC Dale, LB Firth, IP Smith), pp. 105–236. Boca Raton, FL: CRC Press. (doi:10.1201/9780429454455-3)

[7] Kaiser S *et al.* 2013 Patterns, processes and vulnerability of Southern Ocean benthos: a decadal leap in knowledge and understanding. Mar. Biol. 160, 2295–2317. (doi:10.1007/s00227-013-2232-6)

[8] Somero GN . 2010 The physiology of climate change: how potentials for acclimatization and genetic adaptation will determine ‘winners’ and ‘losers’. J. Exp. Biol. 213, 912–920. (doi:10.1242/jeb.037473) 20190116

[9] Ingels J *et al.* 2021 Antarctic ecosystem responses following ice-shelf collapse and iceberg calving: science review and future research. WIREs Clim. Change 12, e682. (doi:10.1002/wcc.682)

[10] Deppeler SL , Davidson AT. 2017 Southern Ocean phytoplankton in a changing climate. Front. Mar. Sci. 4, 40. (doi:10.3389/fmars.2017.00040)

[11] Griffiths HJ , Meijers AJS, Bracegirdle TJ. 2017 More losers than winners in a century of future Southern Ocean seafloor warming. Nat. Clim. Change 7, 749–754. (doi:10.1038/nclimate3377)

[12] Krumhardt KM , Landrum L, Şen B, DuVivier AK, Levy MN, Nissen C, Holland MM, Jenouvrier S. 2026 Emergent climate change signals within Antarctic sea ice and associated ecosystems. Nat. Clim. Change 16, 364–371. (doi:10.1038/s41558-026-02561-9)

[13] Griffiths HJ , Cummings VJ, Van De Putte A, Whittle RJ, Waller CL. 2024 Antarctic benthic ecological change. Nat. Rev. Earth Environ. 5, 645–664. (doi:10.1038/s43017-024-00583-5)

[14] Sweetman AK *et al.* 2017 Major impacts of climate change on deep-sea benthic ecosystems. Elementa: Sci. Anthropocene 5, 4. (doi:10.1525/elementa.203)

[15] Torre L , Alurralde G, Lagger C, Abele D, Schloss IR, Sahade R. 2021 Antarctic ascidians under increasing sedimentation: physiological thresholds and ecosystem hysteresis. Mar. Environ. Res. 167, 105 284. (doi:10.1016/j.marenvres.2021.105284) 33730611

[16] Braeckman U , Soetaert K, Pasotti F, Quartino ML, Vanreusel A, Saravia LA, Schloss IR, Van Oevelen D. 2024 Glacial melt impacts carbon flows in an Antarctic benthic food web. Front. Mar. Sci. 11, 1 359 597. (doi:10.3389/fmars.2024.1359597)

[17] Brenke N . 2005 An epibenthic sledge for operations on marine soft bottom and bedrock. Mar. Technol. Soc. J. 39, 10–21. (doi:10.4031/002533205787444015)

[18] Di Franco D , Linse K, Griffiths HJ, Brandt A. 2021 Drivers of abundance and spatial distribution in Southern Ocean peracarid crustacea. Ecol. Indic. 128, 107 832. (doi:10.1016/j.ecolind.2021.107832)

[19] Assis J , Tyberghein L, Bosch S, Verbruggen H, Serrão EA, De Clerck O. 2018 Bio-ORACLE v2.0: extending marine data layers for bioclimatic modelling. Global Ecol. Biogeogr. 27, 277–284. (doi:10.1111/geb.12693)

[20] Clarke K , Gorley RN. 2006 Primer v6: user manual/tutorial. Plymouth, UK: PRIMER-E.

[21] Kassambara A . 2020 ggpubr: ‘ggplot2’ based publication ready plots. https://CRAN.R-project.org/package=ggpubr.

[22] Lepš J , Šmilauer P. 2003 Multivariate analysis of ecological data using CANOCO. Cambridge, UK: Cambridge University Press. (doi:10.1017/CBO9780511615146)

[23] Legendre P , Gallagher ED. 2001 Ecologically meaningful transformations for ordination of species data. Oecologia 129, 271–280. (doi:10.1007/s004420100716) 28547606

[24] Scrucca L . 2004 qcc: an R package for quality control charting and statistical process control. R News 4, 7.

[25] Oksanen J *et al.* 2013 Vegan: community ecology package. R Package Version. 2.0-10. https://CRAN.R-project.org/package=vegan (accessed 15 October 2021).

[26] Di Franco D , Brandt A, Linse K. 2021 Macrobenthic Isopoda collected by epibenthic sledge from the southern Weddell Sea in 2012, South Orkney Islands in 2016, Prince Gustav Channel in 2018 and Eastern Antarctic Peninsula in 2019 (Version 1.0) [Data set]. Cambridge, UK: NERC EDS UK Polar Data Centre. (doi:10.5285/68315d6d-e7bf-4da0-a73a-5d6e0ba242a7)

[27] Clark GF , Stark JS, Palmer AS, Riddle MJ, Johnston EL. 2017 The roles of sea-ice, light and sedimentation in structuring shallow Antarctic benthic communities. PLoS ONE 12, e0168391. (doi:10.1371/journal.pone.0168391) PMC5226713 28076438

[28] Gradinger R . 2009 Sea-ice algae: major contributors to primary production and algal biomass in the Chukchi and Beaufort Seas during May/June 2002. Deep-Sea Res. Part II 56, 1201–1212. (doi:10.1016/j.dsr2.2008.10.016)

[29] Jin M , Deal C, Wang J, Alexander V, Gradinger R, Saitoh S, Iida T, Wan Z, Stabeno P. 2007 Ice-associated phytoplankton blooms in the southeastern Bering Sea. Geophys. Res. Lett. 34, L06612. (doi:10.1029/2006GL028849)

[30] Jansen J *et al.* 2018 Abundance and richness of key Antarctic seafloor fauna correlates with modelled food availability. Nat. Ecol. Evol. 2, 71–80. (doi:10.1038/s41559-017-0392-3) 29230028

[31] Vernet M *et al.* 2019 The Weddell Gyre, Southern Ocean: present knowledge and future challenges. Rev. Geophys. 57, 623–708. (doi:10.1029/2018RG000604)

[32] Moore JK *et al.* 2018 Sustained climate warming drives declining marine biological productivity. Science 359, 1139–1143. (doi:10.1126/science.aao6379) 29590043

[33] Lannuzel D *et al.* 2020 The future of Arctic sea-ice biogeochemistry and ice-associated ecosystems. Nat. Clim. Change 10, 983–992. (doi:10.1038/s41558-020-00940-4)

[34] de Baar H , Buma A, Nolting RF, Cadee GC, Jacques G, Treguer PJ, Ocean Ecosystems. 1990 On iron limitation of the Southern Ocean. Mar. Ecol. Prog. Ser. 65, 105–122. (doi:10.3354/meps065105)

[35] Kim S-U , Kim K-Y. 2021 Impact of climate change on the primary production and related biogeochemical cycles in the coastal and sea ice zone of the Southern Ocean. Sci. Total Environ. 751, 141 678. (doi:10.1016/j.scitotenv.2020.141678) 33182005

[36] Gooday AJ . 2002 Biological responses to seasonally varying fluxes of organic matter to the ocean floor: a review. J. Oceanogr. 58, 305–332. (doi:10.1023/A:1015865826379)

[37] Suess E . 1980 Particulate organic carbon flux in the oceans—surface productivity and oxygen utilization. Nature 288, 260–263. (doi:10.1038/288260a0)

[38] Veit-Köhler G , Guilini K, Peeken I, Sachs O, Sauter EJ, Würzberg L. 2011 Antarctic deep-sea meiofauna and bacteria react to the deposition of particulate organic matter after a phytoplankton bloom. Deep-Sea Res. Part II 58, 1983–1995. (doi:10.1016/j.dsr2.2011.05.008)

[39] Lee C , Wakeham SG, I. Hedges J. 2000 Composition and flux of particulate amino acids and chloropigments in equatorial Pacific seawater and sediments. Deep-Sea Res. Part I 47, 1535–1568. (doi:10.1016/S0967-0637(99)00116-8)

[40] Lee C , Wakeham S, Arnosti C. 2004 Particulate organic matter in the sea: the composition conundrum. Ambio 33, 565–575. (doi:10.1579/0044-7447-33.8.565) 15666690

[41] Witte U *et al.* 2003 In situ experimental evidence of the fate of a phytodetritus pulse at the abyssal sea floor. Nature 424, 763–766. (doi:10.1038/nature01799) 12917681

[42] Aberle N , Witte U. 2003 Deep-sea macrofauna exposed to a simulated sedimentation event in the abyssal NE Atlantic: in situ pulse-chase experiments using 13C-labelled phytodetritus. Mar. Ecol. Prog. Ser. 251, 37–47. (doi:10.3354/meps251037)

[43] Sorbe JC . 1999 Deep-sea macrofaunal assemblages within the Benthic Boundary Layer of the Cap-Ferret Canyon (Bay of Biscay, NE Atlantic). Deep Sea Res. Part II 46, 2309–2329. (doi:10.1016/S0967-0645(99)00064-8)

[44] Pfannkuche O . 1993 Benthic response to the sedimentation of particulate organic matter at the BIOTRANS station, 47°N, 20°W. Deep Sea Res. Part II 40, 135–149. (doi:10.1016/0967-0645(93)90010-K)

[45] Mohd Nasir N , Barnes DKA, Wan Hussin WMR. 2024 Benthic functionality under climate-induced environment changes offshore on the Antarctic Peninsula continental shelf. Mar. Environ. Res. 194, 106 341. (doi:10.1016/j.marenvres.2024.106341) 38183736

[46] Brökeland W , Guðmundsson G, Svavarsson J. 2010 Diet of four species of deep-sea isopods (Crustacea: Malacostraca: Peracarida) in the South Atlantic and the Southern Ocean. Mar. Biol. 157, 177–187. (doi:10.1007/s00227-009-1308-9)

[47] Brandt A , Linse K, Ellingsen KE, Somerfield PJ. 2016 Depth-related gradients in community structure and relatedness of bivalves and isopods in the Southern Ocean. Progr. Oceanogr. 144, 25–38. (doi:10.1016/j.pocean.2016.03.003)

[48] Di Franco D , Linse K, Griffiths H, Haas C, Saeedi H, Brandt A. 2020 Abundance and distributional patterns of benthic peracarid crustaceans from the Atlantic sector of the Southern Ocean and Weddell Sea. Front. Mar. Sci. 7, 554663. (doi:10.3389/fmars.2020.554663)

[49] Michel LN , Danis B, Dubois P, Eleaume M, Fournier J, Gallut C, Jane P, Lepoint G. 2019 Increased sea ice cover alters food web structure in East Antarctica. Sci. Rep. 9, 8062. (doi:10.1038/s41598-019-44605-5) PMC6542827 31147605

[50] Convey P , Peck LS. 2019 Antarctic environmental change and biological responses. Sci. Adv. 5, eaaz0888. (doi:10.1126/sciadv.aaz0888) PMC6881164 31807713

[51] McClain CR , Schlacher TA. 2015 On some hypotheses of diversity of animal life at great depths on the sea floor. Mar. Ecol. 36, 849–872. (doi:10.1111/maec.12288)

[52] Rosenzweig ML , Abramsky Z. 1993How are diversity and productivity related? In Species dsiversity in ecological communities (eds Ricklefs RE, Schluter D), pp. 52–65. Chicago, IL: University of Chicago Press.

[53] Pineda-Metz S , Isla E, Gerdes D. 2019 Benthic communities of the Filchner Region (Weddell Sea, Antarctica). Mar. Ecol. Prog. Ser. 628, 37–54. (doi:10.3354/meps13093)

[54] Brandt A *et al.* 2007 First insights into the biodiversity and biogeography of the Southern Ocean deep sea. Nature 447, 307–311. (doi:10.1038/nature05827) 17507981

[55] Lin Y *et al.* 2021 Decline in plankton diversity and carbon flux with reduced sea ice extent along the Western Antarctic peninsula. Nat. Commun. 12, 4948. (doi:10.1038/s41467-021-25235-w) PMC8368043 34400630

[56] Ferreira A , Costa RR, Dotto TS, Kerr R, Tavano VM, Brito AC, Brotas V, Secchi ER, Mendes CRB. 2020 Changes in Phytoplankton communities along the Northern Antarctic Peninsula: causes, impacts and research priorities. Front. Mar. Sci. 7, 848. (doi:10.3389/fmars.2020.576254)

[57] Meredith MP , Nicholls KW, Renfrew IA, Boehme L, Biuw M, Fedak M. 2011 Seasonal evolution of the upper-ocean adjacent to the South Orkney Islands, Southern Ocean: results from a ‘lazy biological mooring’. Deep-Sea Res. Part II 58, 1569–1579. (doi:10.1016/j.dsr2.2009.07.008)

[58] Collins M *et al.* 2013 Long-term climate change: projections, commitments and irreversibility. Cambridge, UK: Cambridge University Press. (doi:10.1017/CBO9781107415324.024)

[59] Golledge NR , Levy RH, McKay RM, Naish TR. 2017 East Antarctic ice sheet most vulnerable to Weddell Sea warming. Geophys. Res. Lett. 44, 2343–2351. (doi:10.1002/2016GL072422)

[60] Parkinson CL . 2019 A 40-y record reveals gradual Antarctic sea ice increases followed by decreases at rates far exceeding the rates seen in the Arctic. Proc. Natl Acad. Sci. USA 116, 14 414–14 423. (doi:10.1073/pnas.1906556116) PMC6642375 31262810

[61] Strass VH , Rohardt G, Kanzow T, Hoppema M, Boebel O. 2020 Multidecadal warming and density loss in the Deep Weddell Sea, Antarctica. J. Clim. 33, 9863–9881. (doi:10.1175/JCLI-D-20-0271.1)

[62] Ohshima KI *et al.* 2013 Antarctic bottom water production by intense sea-ice formation in the Cape Darnley polynya. Nat. Geosci. 6, 235–240. (doi:10.1038/ngeo1738)

[63] Post AL , Galton-Fenzi BK, Riddle MJ, Herraiz-Borreguero L, O'Brien PE, Hemer MA, McMinn A, Rasch D, Craven M. 2014 Modern sedimentation, circulation and life beneath the Amery Ice Shelf, East Antarctica. Cont. Shelf Res. 74, 77–87. (doi:10.1016/j.csr.2013.10.010)

[64] Auscavitch S , Waller R. 2017 Biogeographical patterns among deep sea megabenthic communities across the Drake Passage. Antarct. Sci. 29, 531–543. (doi:10.1017/S0954102017000256)

[65] Puerta P *et al.* 2020 Influence of water masses on the biodiversity and biogeography of deep-sea benthic ecosystems in the North Atlantic. Front. Mar. Sci. 7, 239. (doi:10.3389/fmars.2020.00239)

[66] David B , Saucède T. 2015 Biodiversity of the Southern Ocean. Amsterdam, The Netherlands: ISTE Press. (doi:10.1016/B978-1-78548-047-8.50005-9)

[67] Griffiths HJ , Arango CP, Munilla T, McInnes SJ. 2011 Biodiversity and biogeography of Southern Ocean pycnogonids. Ecography 34, 616–627. (doi:10.1111/j.1600-0587.2010.06612.x)

[68] Munilla T , Soler Membrives A. 2009 Check-list of the pycnogonids from Antarctic and sub-Antarctic waters: zoogeographic implications. Antarct. Sci. 21, 99–111. (doi:10.1017/S095410200800151X)

[69] Hodapp D *et al.* 2023 Climate change disrupts core habitats of marine species. Global Change Biol. 29, 3304–3317. (doi:10.1111/gcb.16612) 36789726

[70] Brandt A . 2000 Hypotheses on Southern Ocean peracarid evolution and radiation (Crustacea, Malacostraca). Antarct. Sci. 12, 269–275. (doi:10.1017/S095410200000033X)

